# Breast milk immune composition varies during the transition stage of lactation: characterization of immunotypes in the MAMI cohort

**DOI:** 10.3389/fnut.2023.1252815

**Published:** 2023-11-23

**Authors:** Karla Rio-Aige, Aina Fernández-Bargalló, Esteban Vegas-Lozano, Antonio Miñarro-Alonso, Margarida Castell, Marta Selma-Royo, Cecilia Martínez-Costa, Maria José Rodríguez-Lagunas, Maria Carmen Collado, Francisco José Pérez-Cano

**Affiliations:** ^1^Physiology Section, Department of Biochemistry and Physiology, Faculty of Pharmacy and Food Science, University of Barcelona (UB), Barcelona, Spain; ^2^Nutrition and Food Safety Research Institute (INSA-UB), Santa Coloma de Gramenet, Spain; ^3^Department of Genetics, Microbiology and Statistics, Faculty of Biology, University of Barcelona, Barcelona, Spain; ^4^CIBERFES, ISCIII, Madrid, Spain; ^5^Institute of Agrochemistry and Food Technology (IATA-CSIC), National Research Council, Paterna, Spain; ^6^Department of Pediatrics, INCLIVA Biomedical Research Institute, University of Valencia, Valencia, Spain

**Keywords:** breastfeeding, breast milk, transitional stage, immunoglobulins, cytokines, adipokines

## Abstract

**Background:**

Breast milk is a complex and dynamic fluid needed for infant development and protection due to its content of bioactive factors such as immunoglobulins (Igs). Most studies focus primarily on IgA, but other types of Ig and even other immune components (cytokines and adipokines) may also play significant roles in neonatal health. As a first step, we aimed to characterize the Ig profile, many cytokines, and two adipokines (leptin and adiponectin) at two sampling time points within the transitional stage, which is the least studied phase in terms of these components. The secondary objective was to identify different breast milk immunotypes in the MAMI cohort substudy, and finally, we further aimed at analyzing maternal and infant characteristics to identify influencing factors of breast milk immune composition.

**Methods:**

Breast milk samples from 75 mothers were studied between days 7 and 15 postpartum. The Igs, cytokines, and adipokine levels were determined by a multiplex approach, except for the IgA, IgM, and leptin that were evaluated by ELISA.

**Results:**

IgA, IgM, IgE, IgG2, IL-1β, IL-5, IL-6, IL-10, and IL-17 were significantly higher on day 7 with respect to day 15. The multiple factor analysis (MFA) allowed us to identify two maternal clusters (immunotypes) depending on the breast milk immune profile evolution from day 7 to day 15, mainly due to the IgE and IgG subtypes, but not for IgA and IgM, which always presented higher levels early in time.

**Conclusion:**

All these results demonstrated the importance of the dynamics of the breast milk composition in terms of immune factors because even in the same lactation stage, a difference of 1 week has induced changes in the breast milk immune profile. Moreover, this immune profile does not evolve in the same way for all women. The dynamic compositional changes may be maternal-specific, as we observed differences in parity and exclusive breastfeeding between the two BM immunotype groups, which could potentially impact infant health.

## 1 Introduction

Breast milk (BM) is considered the gold-standard food for infants since, in addition to the basic nutrients (carbohydrates, lipids, proteins, vitamins, and minerals), it also contains a huge variety of bioactive compounds, including growth factors and anti-infective molecules such as immunoglobulins (Ig), cytokines (CKs), and human milk oligosaccharides. All of these components contribute to the proper growth of the baby, both anatomically and neurologically, while also providing defenses and collaborating in the microbiota colonization and the immunity development of the offspring (1–3).

Among other anti-infective compounds in BM, Igs are the most studied, mainly IgA (1), whose presence has been known for a long time (4–12). Although less information is available about the presence of other Igs (IgM, IgG, and IgE), they have been gaining attention in the past 10 years (13) because they can also be influenced by maternal factors and infant requirements. It is reported that an infant and/or maternal infection induces an increase in secretory IgA (sIgA) and IgG in human BM (14). Among these bioactive compounds, CKs, which are in low concentrations (15), have also been generating considerable interest in recent years. Detectable CKs in BM are mainly transforming growth factor (TGF)-β, interleukin (IL)-6, IL-1β, tumor necrosis factor (TNF)-α, IL-10, IL-5, IL-4, IL-13, IL-12, granulocyte colony-stimulating factor (G-CSF), granulocyte-macrophage colony-stimulating factor (GM-CSF), and macrophage colony-stimulating factor (M-CSF) (16–18). Moreover, adipokines can also be found in BM, such as leptin and adiponectin, which are involved in fetal development and growth (19–23) and have immunomodulatory actions (24). In this regard, leptin and adiponectin regulate both innate and adaptive immune responses (25, 26).

It is well known that BM components change along the phases of breastfeeding, leading to three different types of milk: colostrum (from birth to 5–6 days), transitional milk (from 7 to 15 days), and mature milk (since 15 days), with colostrum being the richest in immunological components (13, 27). It is important to highlight that the transition stage is the least studied in terms of the immune composition of BM, mainly for IgG, their subtypes, and many cytokines and adipokines. In addition to compositional changes due to the time of milk sampling (18, 28–33), BM composition can be influenced by maternal prenatal and postnatal factors including diet (34, 35), vaccination (36–39), geographic location, antibiotics (40), smoking (41), maternal pathologies and infections (42), and maternal psychological stress (43). BM composition also seems to be associated with gestational age (31, 44, 45). The BM of mothers of preterm neonates has immune compensatory mechanisms to accelerate their development, such as increased concentrations of IL-6, TGF-β1, and TGF-β2 (31) and IgA, IgM, and IgG (31, 44, 46). Moreover, BM components also change by neonatal requirements during infant infections (14, 47).

In this study, we first aimed to characterize the BM Ig and immune-related compounds, including CK and growth factors, at two sampling time points within the transitional stage. The secondary objective was to identify immunological profiles in BM. Finally, we aimed to identify associations among maternal and infant characteristics able to influence BM immune composition.

## 2 Materials and methods

### 2.1 Cohort and study subjects

The present study was performed in a subgroup of 75 healthy mother–infant pairs within the MAMI birth cohort, which is a clinical prospective mother–infant birth cohort from the Mediterranean area (48) (Clinical Trial Registry NCT03552939). The subgroup of the MAMI cohort was chosen based on breast milk sample availability at both time points (days 7 and 15) within the transition stage. Women with accessible milk samples who adhered to exclusive breastfeeding practices at the specified time points (7 and 15 days) were considered for inclusion. Our study participants were in good health, with no reported chronic health conditions. Exclusion criteria encompassed non-compliance with any of the inclusion criteria, the use of medications or drugs, the presence of chorioamnionitis, mastitis, or health complications during the gestational and breastfeeding periods, and the presence of any chronic diseases or medication usage for chronic conditions. All mothers filled out a clinical questionnaire about them and their children throughout the study (49). Maternal and neonatal clinical records, including mode of birth, neonate gender, maternal BMI, antibiotic exposure, and others, were recorded. The weight and height of the mothers and the infants were measured in medical consultation at different time points: during pregnancy, at birth, at 7 and 15 days, and at 1, 6, 12, 18, and 24 months postpartum.

Furthermore, the mothers also filled out a 140-item Food Frequency Questionnaire (FFQ) about their regular diet during pregnancy (50). The FFQ information was analyzed to obtain data on the intakes of macronutrients and micronutrients per day. For that, FFQ was previously validated in this same cohort by a 3-day questionnaire (51). The analysis of this information permitted the identification of two different dietary patterns in the MAMI cohort: Diet I and Diet II groups, by clustering using the Jensen–Shannon distance and partitioning around medoid clustering methods (51). Diet I was characterized by a higher intake of total dietary fiber, vegetable protein, and polyunsaturated fatty acids, while Diet II was based on a higher intake of animal protein and saturated fatty acids.

Moreover, 72 of the 75 mothers had their secretor status analyzed. For this, total DNA was obtained as described previously (51) and used to test the secretor status by FUT2 genotyping with the polymerase chain reaction (PCR)-random fragment length polymorphisms (RFLPs) as detailed elsewhere (52). Secretor mothers were those with the positive detection of the FUT2 gene, which encodes for the galactoside 2-alpha-L-fucosyltransferase 2.

### 2.2 Collection of human milk samples

Human milk samples for this study were obtained between 7 and 15 days after birth. All the mothers were asked to collect the samples in a specific way: in the morning before lactation, cleaning the breast skin with a solution of 0.5% chlorhexidine, and using a sterile pumper. In addition, the first 2–3 drops were discarded to normalize the collection, and approximately 10 ml were collected. Milk samples were sent to the biobank and kept at −80°C until the lactic serum was obtained by centrifugation at 2,000 × *g* for 15 min at 4°C. Finally, lactic serum was frozen again at −80°C in different aliquots to carry out all the determinations in one freeze/thaw cycle.

### 2.3 Determination of immunoglobulin, cytokine, and adipokine concentrations

The quantification of Igs (IgE, IgG1, IgG2, IgG3, IgG4), CKs (GM-CSF, IFN-γ, IL-1β, IL-2, IL-4, IL-5, IL-6, IL-9, IL-10, IL-12p70, IL-13, IL-17A, IL-18, IL-21, IL-22, IL-23, IL-27, TNF-α), and adiponectin was performed by ProcartaPlex™ Multiplex immunoassay (Thermo Fisher Scientific, Vienna, Austria) using an Antibody Isotyping 7-Plex Human ProcartaPlex™ panel, a Th1/Th2/Th9/Th17 Cytokine 18-Plex Human ProcartaPlex™ panel, and an Adiponectin Human ProcartaPlex™ Simplex Kit, respectively, following the manufacturer’s instructions as in previous studies (49, 53–58). The plates were run on a Luminex instrument and analyzed in ProcartaPlex Analyst Software (MAGPIX® analyzer, Luminex Corporation) at the Flow Cytometry Unit of the Scientific and Technological Centers of the University of Barcelona (CCiT-UB). Assay sensitivity was as follows: 2.11 ng/ml for IgG1; 16.07 ng/ml for IgG2; 0.08 ng/ml for IgG3; 0.56 ng/ml for IgG4; 0.003 ng/ml for IgE 1.2 pg/ml for GM-CSF; 0.2 pg/ml for IFN-γ; 0.2 pg/ml for IL-1β; 0.8 pg/ml for IL-2; 1.5 pg/ml for IL-4; 0.3 pg/ml for IL-5; 0.4 pg/ml for IL-6; 0.5 pg/ml for IL-9; 0.1 pg/ml for IL-10; 0.04 pg/ml for IL-12p70; 0.1 pg/ml for IL-13; 0.1 pg/ml for IL-17A; 0.4 pg/ml for IL-18; 0.6 pg/ml for IL-21; 8.2 pg/ml for IL-22; 0.9 pg/ml for IL-23; 5.1 pg/ml for IL-27; 0.4 pg/ml for TNF-α; 4.6 pg/ml for adiponectin.

The quantification of leptin was performed by a Quantikine® Colorimetric Sandwich ELISA Kit (R&D Systems, Minneapolis, MN, USA) following the manufacturer’s instructions as in previous studies (41). Data were analyzed by Multiskan Ascent v2.6 software (Thermo Fisher Scientific, Vienna, Austria). Assay sensitivity was 7.8 pg/ml.

The quantification of IgA and IgM was performed by an ELISA kit from Bethyl Laboratories (Montgomery, TX, USA) and an ELISA kit from Cloud-Clone Corp. (Houston, TX, USA), respectively, following the manufacturer’s instructions as in previous studies (31, 59). These kits included the quantification of the secretory form of both types of immunoglobulins. Assay sensitivity was 14.1 ng/ml for IgM, and 1.03–750 ng/ml was the assay range for IgA. Data were analyzed by Multiskan Ascent v2.6 software (Thermo Fisher Scientific, Vienna, Austria).

### 2.4 Data processing and statistical analysis

The results are expressed as mean ± SEM (standard error of the mean) unless otherwise specified. Box plots showed the interquartile range, with the error bars representing the lowest and highest values. Shapiro–Wilk and Levene’s tests were used to determine the normality and homogeneity of the data variance, respectively. When variables were not normally distributed, a logarithmic normalization of the data was made or non-parametric tests were used. Multivariate general linear model (GLM) and repeated measures GLM analyses were conducted to assess the variance in multiple dependent variables using two-factor variables across measurements taken at different time points. For example, this approach was applied to analyze the tracking of infant weight and height up to the first month of life in the two study groups (BM immunotypes). Spearman’s correlation coefficient was used to search for correlation between variables that were not normalized. Student’s t-test and Mann–Whitney U-test were used to assess significant differences between groups, while the chi-square test (X^2^) compared frequencies, such as the detectability of CKs. For cytokines, a left-censored Tobit model with a threshold of 0 was fitted to test differences between times using the R software package AER (60). The *p*-values were adjusted for multiple comparisons using the false discovery rate (FDR) correction. A *p*-value of <0.05 was considered significant in all the tests.

The MFA was performed to integrate the two measurements on days 7 and 15 with the aim of providing a comprehensive understanding of the contribution of the different variables and times to the variability of the data. The MFA models were graphically displayed by plotting the projections of the samples onto the bidimensional space defined by the two first dimensions. All the data were normalized by means of log transformation prior to carrying out these statistical analyses using the R software packages FactoMineR and factoextra (61).

To discover the relationships between milk composition variables and immunotype groups, a mixed graphical model (MGM) was used. MGMs are undirected probabilistic graphical models, where each node corresponds to one variable, and the edges between two nodes represent a conditional dependency between them given all other variables in the graphical model (62). We have used ‘mgm’ R-package to estimate the network of dependencies (63). Specifications were set to allow the maximum number of interactions in the network.

All statistical analyses were performed using IBM SPSS Statistics 22 (IBM, USA) and R version 4.1.2 (R Foundation, Austria) (64).

## 3 Results

### 3.1 Study population

The mean maternal weight gain during pregnancy was in the range recommended by the Institute of Medicine (65). Overall, approximately 50% of them used antibiotics during pregnancy and/or on delivery day. More than half of the participants’ deliveries were vaginal, and approximately 50% of the total participants were first-time mothers (Table 1). The table includes the breast milk (BM) immunotype classification, depending on the later results.

**Table 1:** Mother characteristics.

Maternal characteristics	ALL	*N*	BM-I	*N*	BM-II	*N*	*p*-value
Pre-gestational BMI (Kg/m^2^),^1^ mean ± SEM	22.00 ± 0.40	75	23.20 ± 0.64	39	22.00 ± 0.40	36	0.286
Pregnancy weight gain (Kg),^1^ mean ± SEM	11.80 ± 0.69	75	11.86 ± 0.71	39	11.80 ± 0.69	36	0.966
Antibiotic during pregnancy,^2^ yes (%)	24 (32.00)	75	15 (38.46)	39	15 (41.67)	36	0.211
Intrapartum antibiotic,^2^ yes (%)	29 (38.67)	75	14 (35.90)	39	9 (25)	36	0.608
^a^Perinatal antibiotic,^2^ yes (%)	42 (56.00)	75	22 (56.41)	39	20 (55.55)	36	0.940
Gestational age (weeks),^1^ mean ± SEM	39.33 ± 0.22	75	39.73 ± 0.19	39	39.33 ± 0.22	36	0.177
Mode of delivery: vaginal birth,^2^ yes (%)	49 (65.33)	75	26 (66.67)	39	23 (63.89)	36	0.801
Primipara,^2^ yes (%)	41 (54.67)	75	16 (41.02)	39	25 (69.44)^*^	36	0.014
Animals,^2^ yes (%)	17 (26.98)	63	8 (25.81)	31	9 (28.13)	32	0.836
Exclusive maternal breastfeeding until 15th day,^2^ yes (%)	64 (85.33)	75	37 (94.87)	39	27 (75.00)^*^	36	0.015
Exclusive maternal breastfeeding until 6-month,^2^ yes (%)	57 (76.00)	75	33 (84.62)	39	24 (66.67)	36	0.069
Diet I cluster,^2^ yes (%)	28 (37.33)	75	12 (30.77)	39	16 (44.44)	36	0.221
Secretor mother,^2^ yes (%)	55 (76.39)	72	25 (71.43)	35	30 (81.10)	37	0.335

Maternal characteristics associated with the total and with the BM immunotype groups. BM-I, breast milk immunotype I; BM-II, breast milk immunotype II; BMI, body mass index; SEM, standard error of the mean. Exclusive maternal breastfeeding refers to the provision of human milk and excludes the use of formula milk.

1 *p*-values were calculated with Student’s t-test.

2 *p*-values were calculated using X^2^test.

* *p* < 0.05 comparing two BM immunotype groups.

a Perinatal antibiotic includes both antibiotics during pregnancy and/or intrapartum antibiotics.

With regard to the infant characteristics, slightly more than half of the infants were females, 23% took antibiotics from birth to 2 years, and 11 of 32 infants developed atopy. As expected, the intake of antibiotics and the number of infections increased over time (Table 2).

**Table 2:** Infant characteristics.

Infant characteristics	ALL	*N*	BM-I	*N*	BM-II	*N*	*p*-value^1^^,^^2^	*p*-value^3^
Gender: Female,^2^ yes (%)	42 (56.00)	75	21 (53.85)	39	21 (58.33)	36	0.696	
Antibiotic intake,^2^ yes (%)								
0–1 month	5 (6.67)	75	3 (7.69)	39	2 (5.55)	36	0.711	
0–6	9 (12.33)	73	6 (16.22)	37	3 (8.33)	36	0.306	
0–12	17 (26.56)	64	11 (34.38)	32	6 (18.75)	32	0.157	
0–24	23 (71.88)	32	14 (87.50)	16	9 (56.25)^*^	16	0.049	
Atopy (0–24 month),^2^ yes (%)	11 (34.38)	32	5 (31.25)	16	6 (37.50)	16	0.710	
^a^Number of infections,^1^ mean ± SEM								
0–6 months	0.42 ± 0.13	73	0.38 ± 0.11	37	0.42 ± 0.13	36	0.827	
0–12 months	0.72 ± 0.20	72	0.61 ± 0.13	36	0.72 ± 0.20	36	0.648	
0–24 months	6.82 ± 0.85	33	8.00 ± 1.43	16	6.82 ± 0.85	17	0.485	
Weight (kg),^1^ mean ± SEM								Group: 0.033
Birth	3.15 ± 0.08	75	3.29 ± 0.07	39	3.15 ± 0.08	36		Time: <0.001
7th day	3.08 ± 0.06	72	3.31 ± 0.07	39	3.08 ± 0.06	34		Int: 0.76
15th day	3.33 ± 0.07	71	3.57 ± 0.08	37	3.33 ± 0.07	34		
1st month	3.98 ± 0.09	73	4.16 ± 0.09	37	3.98 ± 0.09	36		
Height (cm),^1^ mean ± SEM								Group: 0.01
Birth	49.26 ± 0.41	75	50.08 ± 0.35	39	49.26 ± 0.41	36		Time: <0.001
7th day	49.73 ± 0.32	72	51.24 ± 0.34	39	49.73 ± 0.32	34		Int: 0.52
15th day	51.10 ± 0.35	71	52.30 ± 0.37	37	51.10 ± 0.35	34		
1st month	53.44 ± 0.40	73	54.61 ± 0.40	37	53.44 ± 0.40	36		
BMI z-score,^1^ mean ± SEM								Group: 0.46
Birth	−0.46 ± 0.15	75	−0.29 ± 0.15	39	−0.46 ± 0.15	36		Time: 0.030
7th day	−0.65 ± 0.13	72	−0.55 ± 0.16	39	−0.65 ± 0.13	34		Int: 0.59
15th day	−0.70 ± 0.15	71	−0.47 ± 0.16	37	−0.70 ± 0.15	34		
1st month	−0.66 ± 0.11	73	−0.65 ± 0.20	37	−0.66 ± 0.11	36		

Maternal characteristics associated with the total and BM immunotype groups. BM-I, breast milk immunotype I; BM-II, breast milk immunotype II; BMI, body mass index; SEM, standard error of the mean.

1 *p*-values were calculated with Student’s t-test comparing two BM immunotype groups.

2 *p*-values were calculated using a X^2^test comparing the two BM immunotype groups.

3 *p*-values were calculated using repeated measures GLM for the BM immunotype group variable, the time variable, and the interaction (Int) between these two independent variables.

* *p* < 0.05.

a Number of infections includes both respiratory and gastrointestinal.

### 3.2 Changes in the immune composition of breast milk from day 7 to day 15

The description of some immune components, particularly the characterization of the IgG isotype profile and IgE levels found in transition milk, is described for the first time in this study. The immune compounds present in milk on days 7 and 15 of lactation were analyzed and compared.

**Figure 1:**
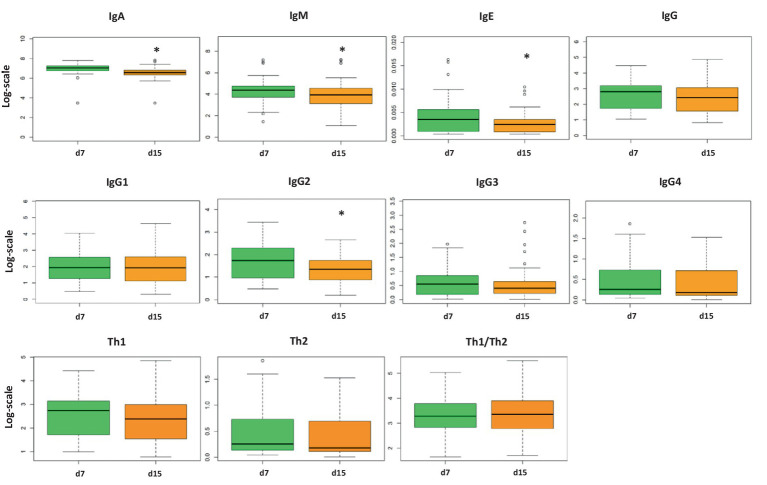
Concentration of immunoglobulin (Ig) levels in breast milk on days 7 and 15 from the same mothers (*N* = 75). The concentration of each analysis (pg/ml) was normalized logarithmically, and the mean from each group is expressed in box plots (interquartile range). Student’s t-test was used to determine significant differences between sampling days. The p-values were adjusted for multiple comparisons using the FDR correction. *p < 0.05. IgG1, IgG2, and IgG3 (Igs associated with Th1 response); IgG4 (Ig associated with Th2 response).

Although there were no differences in adipokine levels (Supplementary Figure 1), the immunoglobulin and cytokine concentrations showed some differences between days 7 and 15 (Figure 1; Table 3, respectively). IgA, IgM, and IgE showed their highest values on day 7. However, IgG levels did not change between milk from day 7 to day 15, and with respect to the IgG isotypes associated with the Th1 or Th2 response, only IgG2 displayed a significant decrease with time (*p* < 0.05) even though it did not affect the Th1/Th2 ratio. With regard to the cytokine levels, day 7 presented a higher concentration of IL-1β, IL-6, IL-9, IL-10, and IL-17 (*p* = 0.021, *p* = 0.020, *p* = 0.0021, *p* = 0.020, and *p* = 0.048, respectively) than day 15. On the contrary, IFN-γ and IL-18 levels were higher on day 15 than on day 7 (*p* = 0.021 and *p* = 0.028, respectively). Overall, the cytokine concentration in the samples analyzed was variable, and some cytokines were not detected in some subjects, similar to what has been previously described (66) (Table 3). The components were grouped depending on their functions to study the immune response (pro-inflammatory, anti-inflammatory, Th1, Th2, Th9, Th17, hematopoietic factors, innate immunity, and acquired immunity) (27, 58, 67–75) (Supplementary Figure 2). Some of the calculated variables were also significantly higher on day 7 than on day 15, such as anti-inflammatory response, Th2, Th17, innate immunity, and acquired immunity (Supplementary Figure 3).

**Table 3:** Concentrations and detectability of cytokines in breast milk.

Cytokines	d7, *N* = 75			d15, *N* = 75		
	pg/ml	%det	IQR	pg/ml	%det	IQR
GM-CSF	0.07 ± 0.03	6.67 (5/75)	0.00–0.00	0.01 ± 0.01	1.33 (1/75)	0.00–0.00
IFN-γ	0.08 ± 0.03	20 (15/75)	0.00–0.00	0.13 ± 0.03^*^	21.33 (16/75)	0.00–0.00
IL-1β	0.32 ± 0.06	45.33 (34/75)	0.00–0.56	0.19 ± 0.05^*^	26.67 (20/75)^*^	0.00–0.08
IL-2	0.14 ± 0.04	13.33 (10/75)	0.00–0.00	0.03 ± 0.02	4 (3/75)^*^	0.00–0.00
IL-4	0.04 ± 0.02	6.67 (5/75)	0.00–0.00	0.00 ± 0.00	1.33 (1/75)	0.00–0.00
IL-5	0.09 ± 0.03	13.33 (10/75)	0.00–0.00	0.06 ± 0.03	6.67 (5/75)	0.00–0.00
IL-6	0.92 ± 0.10	61.33 (46/75)	0.00–1.74	0.60 ± 0.09^*^	45.33 (34/75)^*^	0.00–1.31
IL-9	0.08 ± 0.03	14.67 (11/75)	0.00–0.00	0.03 ± 0.01	12 (9/75)	0.00–0.00
IL-10	0.14 ± 0.03	45.33 (34/75)	0.00–0.16	0.11 ± 0.03^*^	45.33 (34/75)	0.00–0.06
IL-12	0.06 ± 0.02	33.33 (25/75)	0.00–0.04	0.04 ± 0.01	45.33 (34/75)	0.00–0.06
IL-13	0.01 ± 0.01	1.33 (1/75)	0.00–0.00	0.01 ± 0.01	2.67 (2/75)	0.00–0.00
IL-17	0.09 ± 0.03	16 (12/75)	0.00–0.00	0.03 ± 0.02^*^	8 (6/75)	0.00–0.00
IL-18	0.94 ± 0.06	94.67 (71/75)	0.53–1.32	1.09 ± 0.07^*^	92 (69/75)	0.73–0.74
IL-21	0.64 ± 0.06	85.33 (64/75)	0.22–0.97	0.56 ± 0.05	85.33 (64/75)	0.31–0.74
IL-22	0.68 ± 0.06	74.67 (56/75)	0.00–1.14	0.66 ± 0.06	74.67 (56/75)	0.00–1.03
IL-23	0.07 ± 0.03	8 (6/75)	0.00–0.00	0.03 ± 0.01	4 (3/75)	0.00–0.00
IL-27	0.03 ± 0.02	2.67 (2/75)	0.00–0.00	0.03 ± 0.03	1.33 (1/75)	0.00–0.00
TNF-α	0.41 ± 0.04	88 (66/75)	0.14–0.58	0.35 ± 0.03	85.33 (64/75)	0.14–0.52

Data show the concentration of cytokines (CKs) in breast milk on day 7 and day 15 from the same mothers (*N* = 75). The concentration of each analysis (pg/ml) was normalized logarithmically and expressed as mean ± S.E.M, detectability frequencies (%det), and interquartile ranges (IQRs). Tobit model was used to determine significant differences between sampling days. X^2^ test compared detectability. The p-values were adjusted for multiple comparisons using the FDR correction. GM-CSF, granulocyte macrophage colony-stimulating factor; IFN, interferon; IL, interleukin; TNF, tumor necrosis factor.

* *p* < 0.05.

### 3.3 Description of BM immunotypes

**Figure 2:**
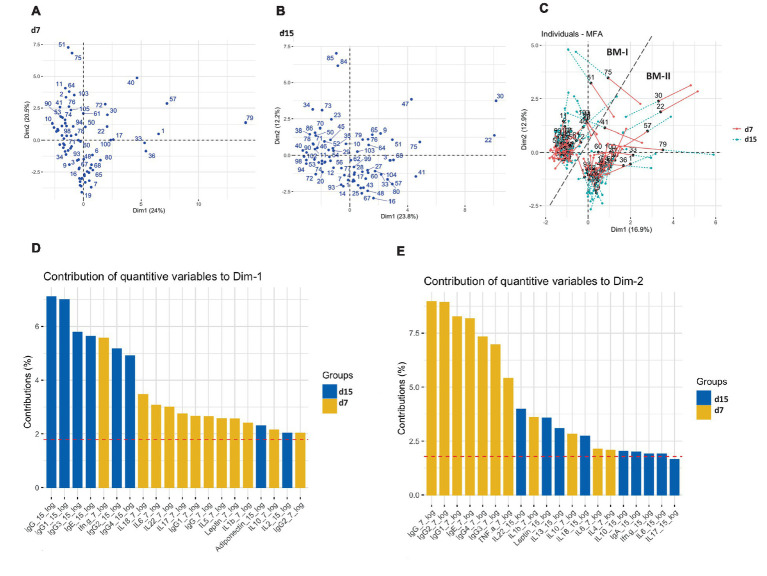
Multiple factor analysis (MFA) for the concentration of immune factors in the breast milk of 75 mothers (MAMI cohort) on days 7 **(A)**, 15 **(B)**, and taking into consideration the two sampling time points **(C)**. The contribution of quantitative variables to both dimensions (Dim): Dim-1 **(D)** and Dim-2 **(E)**.

**Figure 3:**
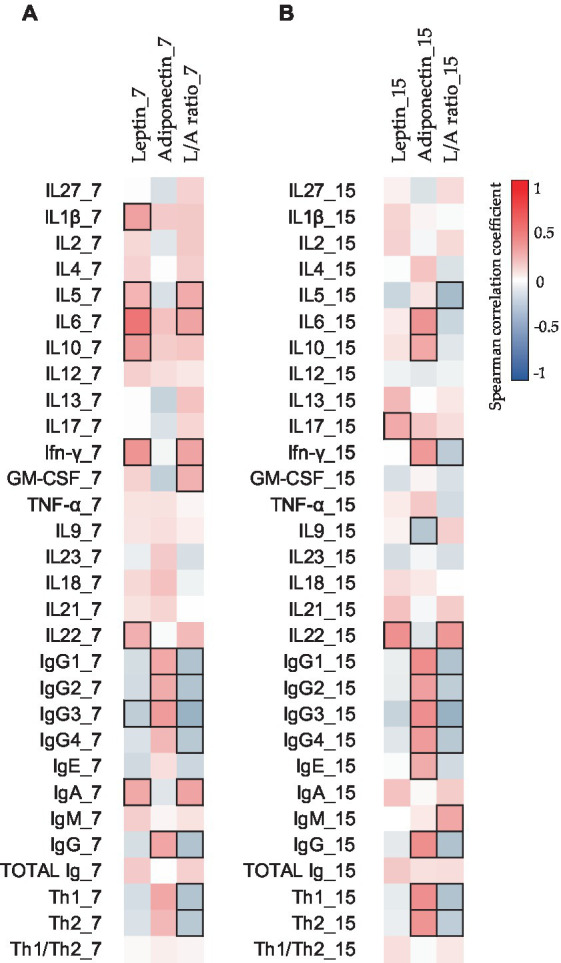
Correlations between the CK and Ig compositions present in BM on days 7 **(A)** and 15 **(B)** with the adipokine composition at the same sampling time. The Spearman correlation coefficient is represented in the heatmap following the color in the legend. Bold frames represent correlations with statistical significance (*p* < 0.05). GM-CSF, granulocyte-macrophage colony-stimulating factor; IFN, interferon; IL, interleukin; L/A ratio, leptin/adiponectin ratio; TNF, tumor necrosis factor. IgG1, IgG2, and IgG3 (Igs associated with Th1 response); IgG4 (Ig associated with Th2 response) (76, 77).

A multivariate factor analysis (MFA) was performed to observe different profiles regarding the BM immune composition. The distribution of the mothers taking into consideration each day separately, on days 7 and 15 (Figures 2A,B, respectively), did not show any particular aggrupation. However, when the MFA was generated considering the two types of milk from the same mother, two clusters were identified with strong similarities among individuals (Figure 2C). It was also observed that the mothers from the same cluster had similar dynamic behavior regarding their BM immune components from d7 to d15. The clusters were based on the BM immune components; therefore, they were named BM immunotype I (BM-I) and BM immunotype II (BM-II). Furthermore, as represented in Supplementary Figure 4, considering each day separately and the existence of the two BM immunotypes, the two clusters could already be observed, but without a good separation, as noticed in Figure 3C.

The next step was to characterize these clusters, or BM immunotypes, to define the differences in composition between them and to study the association with maternal and infant characteristics to find potential sources of variability in the composition of milk immune molecules.

The variables that influenced the most separation among the participants were the Ig levels, mainly IgG subtypes, and total IgG. Observing the importance of the variables in each dimension (Dim) defining the MFA, it can be established that while Dim-1 was described mainly by IgG-15d, IgG1-15d, and IgG3-15d, Dim-2 was linked to levels of IgG-7d, IgG1-7d, and IgG2-7d (Figures 2D,E, respectively). In line with these results, these components displayed differing temporal evolutions when compared between clusters, as was observed in the interaction of variables (group and time) by conducting a multivariate general linear model (GLM; Supplementary Table 1). Additionally, differences between groups were also observed when fixing the sampling time for those components that exhibit distinct temporal evolutions. For instance, the BM immunotype II showed higher levels of IgG, IgG1, and IgG2 on day 15 with respect to the BM immunotype I, even though on day 7 they were higher in the BM immunotype I (Supplementary Table 1). The influence of these components in clustering, and of others such as IgE and IL-22 was confirmed when strong relations were directly found in the mixed graphical model (MGM) with the BM immunotype group (Supplementary Figure 5a). In addition, strong correlations were also observed among the predominant variables that described Dim-2 (IgG1-7d, IgG2-7d, IgG3-7d, IgG4-7d, IgG-7d, and IgE-7d; Supplementary Figure 5b). In addition, IgG1-15d and IgG-15d, which are important in Dim-1 were also highly correlated (Supplementary Figure 5b).

Moreover, to investigate the relations between all the components, Spearman’s correlations were made with the non-transformed data. The study of the impact of adipokine levels on the other BM immune components can be observed in Figure 3. Leptin and adiponectin showed significant positive correlations with several BM CKs and Igs (Figure 3).

**Figure 4:**
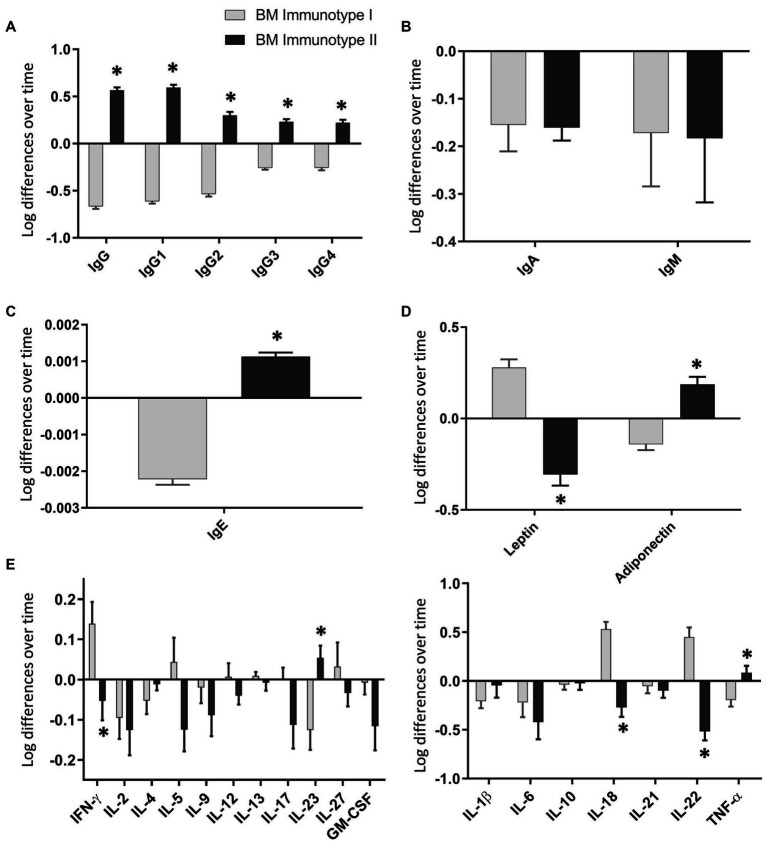
Changes in the breast milk immunoglobulin **(A–C)**, adipokine **(D)**, and cytokine **(E)** levels over time in each breast milk immunotype group. BM-I, breast milk immunotype I (*n* = 39); BM-II, breast milk immunotype II (*n* = 36). The normalized data from day 15 were subtracted from the normalized data from day 7 and are expressed as mean ± SEM. Student’s t-test was used to determine significant differences between groups. The *p*-values were adjusted for multiple comparisons using the FDR correction. *p < 0.05.

To evaluate the dynamic behavior of the BM immune components within the BM immunotype group, we calculated the increase of each immune variable with the transformed data from d7 to d15 (Figure 4). Each BM immunotype group had a different dynamic pattern. Importantly, the most prominent components were the strongest in the dimensions of the MFA. As all the IgG subtype levels increased from d7 to d15 in BM-II, the total IgG concentration was also boosted in the BM-II group (Figure 4A). IgE and adiponectin concentrations also showed the same pattern (Figures 4C,D, respectively). The opposite behavior was observed in the BM-I cluster for the same components. On the contrary, while leptin levels increased after 1 week in the BM-I, they decreased in the BM-II (Figure 4D). Importantly, by assessing the IgA and IgM changes, we found that they behaved the same in both groups. Thus, these Igs decreased from d7 to d15 in all mothers (Figure 4B). In terms of cytokine concentration, IL-18, IL-22, IL-23, IFN-γ, and TNF-α also showed significantly different dynamic behaviors when comparing the two groups. For instance, IL-5, IL-18, IL-22, and IFN-γ increased over time in the BM-I group, while they decreased over time in the BM-II group.

### 3.4 Potential contribution of maternal factors to the breast milk immune composition

To further investigate the potential source of variability between clusters, the maternal characteristics within each group were compared in relation to the BM immunotype. The maternal variables (e.g., pre-gestational BMI, weight gain, the secretory classification, and the presence of animals at home) and the gestational aspects (i.e., gestational age, health complications during pregnancy, and antibiotic use) were similar between the two groups, allowing us to discard these factors as contributors to the clustering of the BM immunotypes. However, mothers belonging to BM-I displayed a lower frequency of being first-time mothers (Table 1) and a tendency to increase the number of mothers practicing exclusive breastfeeding (*p* = 0.069; Table 1) with respect to BM-II mothers. In line with these results, more mothers started a mixed feeding before day 15 in the BM-II group (*p* = 0.015) than in the BM-I group. To investigate the impact of the mother’s genetic background with respect to HMO production in the BM immunotype, it was also possible to classify the mothers according to their secretor gene (secretor mother and non-secretor mother). The results showed that the genetic background did not influence the BM immune composition of the studied mothers (Supplementary Figure 6A).

Mothers were also classified into two dietary groups (Supplementary Figure 6B) according to their dietary records, as done in previous studies with the same cohort and as described in the MM section (51, 78). While the Diet I group ate foods richer in fiber and vegetable protein, the Diet II group ate foods richer in animal protein and saturated fatty acids. Although a clear association of the dietary group with the BM immunotype was not found (Supplementary Figure 6B), plant-derived metabolites, such as total polyphenols, tended to increase in the mothers belonging to the BM-II group (*p* = 0.066; Supplementary Figure 6C); however, vitamin D levels were lower in BM-II with respect to the BM-I group (Supplementary Figure 6D).

### 3.5 Effect of immune components and immunotypes on infant growth and infection incidence

To investigate the impact of having different immune dynamic profiles in the transition stage, the infant characteristics between the two groups (i.e., gender, weight, height, BMI z-scores, atopy, antibiotic use, and number of infections) were also analyzed (Table 2). Although children of mothers belonging to the BM-I group seemed to exhibit higher weight and height during the first month of life, the weight trajectory over time did not differ between clusters (Table 2), and the increase in weight and height from birth to the first month of life is similar when comparing the two groups (data not shown).

**Figure 5:**
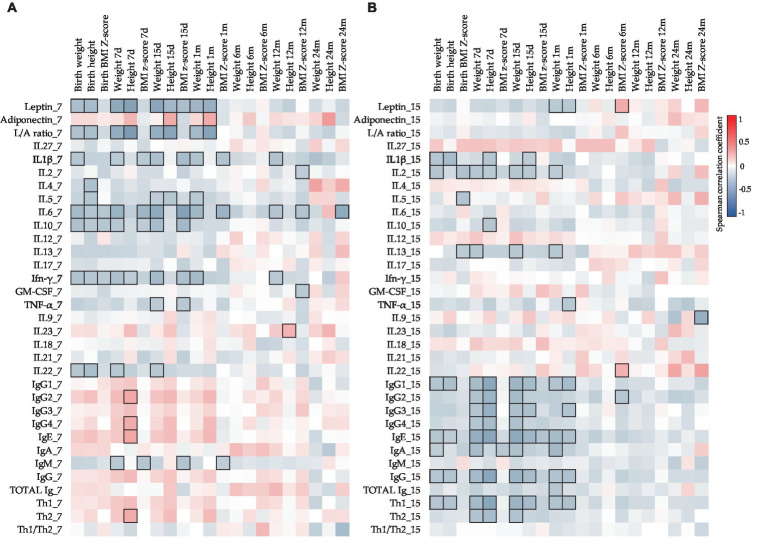
Correlations between CK, Ig, and adipokine levels present in BM on days 7 **(A)** and 15 **(B)** with the infant growth parameters. The Spearman correlation coefficient is represented in the heatmap following the color in the legend. Bold frames represent correlations with statistical significance (*p* < 0.05). GM-CSF, granulocyte-macrophage colony-stimulating factor; IFN, interferon; IL, interleukin; L/A ratio, leptin/adiponectin ratio; TNF, tumor necrosis factor. IgG1, IgG2, and IgG3 (Igs associated with Th1 response); IgG4 (Ig associated with Th2 response) (76, 77).

To assess the influence of each BM immune component on infant growth, the Spearman correlations between these parameters were also studied (Figure 5). Surprisingly, different correlations were found in the two BM sampling days (Figures 5A,B). While negative significant correlations between leptin at 7 days and the weights and heights from birth to 1 month were found (Figure 5A), the leptin levels at 15 days only showed significant negative correlations with the weight and the height at 1 month of age (Figure 5B). Additionally, more correlations between these infant parameters and CKs were found in the BM at 7 days than in the BM at 15 days. Contrary to what happened at 7 days, at 15 days, many important negative correlations were observed between Ig types and infant growth from birth to 1 month (Figure 5B). In addition, it is important to note that most of the correlations with infant parameters were concentrated in the first month of life.

**Figure 6:**
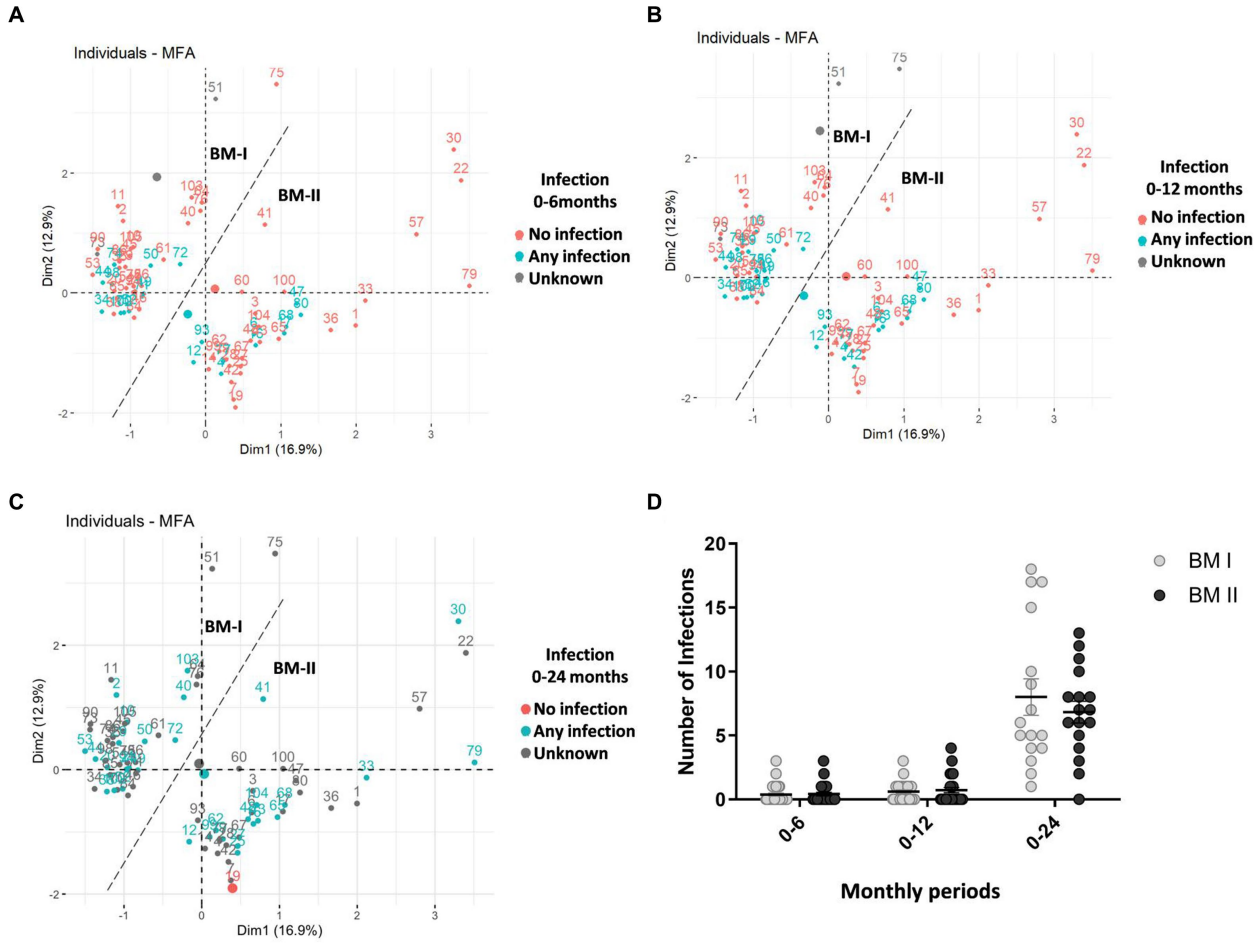
Multiple factor analysis (MFA) for the concentration of immune factors in the breast milk of 75 mothers (MAMI cohort), taking into account the two sampling days (days 7 and 15), considering Dim-1 (x-axis) and Dim-2 (y-axis). In blue, the mothers whose child had any infection, in red, the mothers whose child had no infection, and in gray are the mothers with unknown infection data **(A–C)** from birth to 6 months old **(A)**; from birth to 12 months old **(B)**; from birth to 24 months old **(C)**. The number of total infant infections over the three periods. BM-I, breast milk immunotype I (*n* = 39); BM-II, breast milk immunotype II (*n* = 36); Student’s *t*-test was used to determine significant differences between groups. *p < 0.05 **(D)**.

Continuing with the infant characteristics, the offspring of the BM-I group required more antibiotics from birth to 24 months (Table 1), although it is worth noting that the data decreased throughout the follow-up time. In line with these results, it was also observed that the three children who had more infections from birth to 2 years belonged to the BM-I group (Figure 6D). However, there was no association between the presence of infections and the clusters of BM immunotypes (Figures 6A–C).

## 4 Discussion

The composition of human milk dynamically changes throughout breastfeeding (27, 79). Our results provide the characterization of the Ig profile, 18 cytokines, and 2 adipokines in transitional milk, which is the least studied at the immunological level. Moreover, we demonstrate the presence of different profiles of BM immunotypes for the first time.

In line with other studies, including those from other stages of BM, IgA is the principal Ig found in human milk (80–90% of total Ig) (80, 81), followed by IgM, IgG, and finally IgE (42, 67, 80, 82–87). Indeed, we observed that IgA, IgM, IgG, and IgE were present at a percentage of 91/8/1/0.0001%, respectively, on day 7 and 85/11/4/0.0005%, respectively, on day 15. Little is known about the concentration and impact of the IgG subtypes in BM and even less in the transitional stage; however, they are gaining attention due to the observed functions of IgG in the neonates at an intestinal level in dampening mucosal T helper cell responses and inducing oral tolerance (88–90). As found in the literature, the relative abundances of the IgG subtypes in colostrum and mature milk showed a predominance of IgG1, followed by IgG2, IgG3, and IgG4 (13), in agreement with what we have found in transitional milk in this study. In addition, very few research studies have focused on IgE levels in BM until now, and to our knowledge, this is the first study displaying IgE concentrations in BM during the transition period.

As it has been previously described, transition BM contains a broad variety of cytokines, including IL-1β, IL-2, IL-4, IL-6, IL-8, IL-10, IFN-γ, and TNF-α (18, 66, 91, 92). Moreover, we also found good detectability for IL-18, IL-21, and IL-22 (94.67, 85.33, and 74.67%, respectively), which are cytokines less studied in BM. These CKs may play significant roles in infant immune defenses as they are key players in maintaining intestinal epithelial homeostasis and host defense (93–95). Therefore, they require further attention regarding their concentration in BM and their impact on the neonate. Finally, the cytokines least detected were GM-CSF, IL-4, IL-13, and IL-27, as also observed in other studies (30, 96).

**Figure 7:**
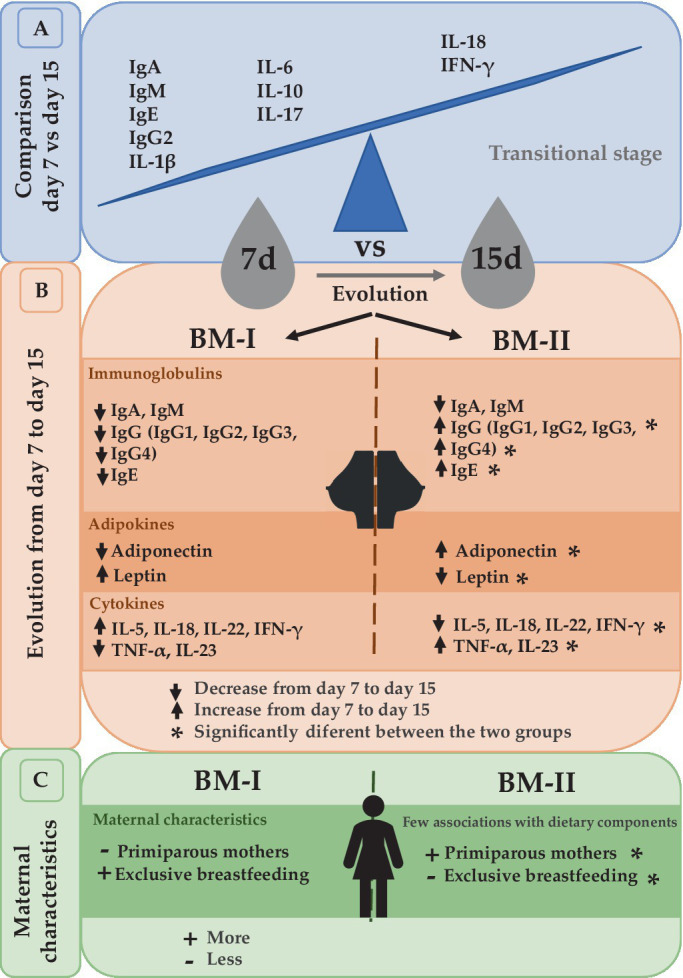
Summary of the comparison between day 7 and day 15 **(A)**. The evolution from day 7 to day 15 led to the separation of the mothers into two groups depending on the dynamical behavior of immunoglobulins, adipokines, and cytokines **(B)**. The maternal characteristics associated with these two clusters **(C)**. BM-I, breast milk immunotype I (*n* = 39); BM-II, breast milk immunotype II (*n* = 36); IFN, interferon; IL, interleukin; TNF, tumor necrosis factor.

It is well established that Ig concentrations, mainly IgA, tend to decrease from colostrum to mature milk (30–32). Furthermore, CK levels also change throughout the lactation period (18, 31). Comparing the BM immune composition from day 7 to day 15, many changes appeared, indicating that BM is a highly dynamic source of these components in the transitional stage, with day 7 being the richest one in immune component levels. In this regard, IgA, IgM, IgE, IgG2, IL-1β, IL-6, IL-10, and IL-17 were significantly higher on day 7 with respect to day 15. However, IFN-γ and IL-18 increased from day 7 to day 15. Overall, these results allow us to conclude that the differences between day 7 and day 15 reinforce the idea that BM is a highly dynamic source of these immune factors, particularly in this period. Moreover, these results highlight the importance of milk collection timing in interventional and descriptive studies, as just a few days lead to dramatically different levels of BM immune components (Figure 7).

After performing the multivariate factor analysis (MFA), two clusters of mothers were observed with different profiles regarding the BM immune composition throughout the transitional milk stage: BM immunotype I (BM-I) and BM immunotype II (BM-II). While the IgA and IgM were not influencing factors in the ordination of the mothers because in both cases their levels decreased from day 7 to day 15, the immune components that drove the separation of the clusters the most were the IgG subtypes. Specifically, IgG1, IgG2, IgG3, and IgG4 increased from day 7 to day 15 in the BM-II mothers and decreased in the BM-I mothers. Furthermore, the same behavior was observed for IgE, IL-23, and TNF-α.

With regard to adipokine levels during breastfeeding, previous studies have reported that leptin levels in BM decrease from colostrum to mature milk. However, our results show that leptin levels were similar on days 7 and 15. When analyzing the data by clustering, we observed that leptin levels increased over time in BM-I but decreased in BM-II. Regarding adiponectin, some studies have shown an increase over time, while others have shown a decrease (97–100). Therefore, we observed that there were groups of mothers with different adiponectin dynamics, which explains the contradictory results in the literature. Furthermore, these adipokines are significant in distinguishing the BM immunotype groups and may have an impact on offspring. In addition, adiponectin levels were positively correlated with IgG1, IgG2, IgG3, and IgG4 on day 7 and with IgG1, IgG2, IgG3, IgG, and IgE on day 15. Consequently, these associations could suggest the potential immunomodulatory role of adipokines in early life, although additional research is required to draw more robust conclusions.

Many maternal and infant variables can impact the composition of breast milk, as observed mainly with IgA, adiponectin, and leptin (14, 31, 82, 101, 102). Further studies are needed to explore more in-depth these influencing factors. The second objective of this study was to explore the variables that could influence the clustering of breast milk into two immunotypes, aiming to identify any variables associated with these distinct milk evolution profiles among mothers. We analyzed the contribution of maternal and infant characteristics in the two clusters and found that there were more first-time mothers and more mixed breastfeeding before the 15th day in BM-II than in BM-I. These two characteristics are related since the breastfeeding experience is different between primiparous mothers and multiparous mothers, since the second ones have more successful initiation of exclusive breastfeeding and are more likely to breastfeed through 6 months (103–105). In addition, parity is a maternal characteristic that seems to have an influence on the BM composition. For instance, it was found that the parity number is positively associated with the lipid concentration but not with the total proteins and sIgA levels in transitional and mature milk samples (106). However, higher levels of IgA, IgG isotypes, IgM, TGF-α, and other factors in colostrum samples from primiparous mothers than from multiparous mothers have been reported (107, 108). In addition, different BM compositions of immune components during the transition stage do not seem to be the strongest factor influencing the infection episodes in early life, but they deserve to be further studied in future. In this line, the maternal diet does not seem to influence the dynamics of the immune composition of the transitional stage BM, contrary to what happens in the case of the infant and BM microbiota (51, 109). Secretor status is critical for HMO composition in BM; however, it does not seem to have an impact on the immune components analyzed here.

Regarding infant characteristics, it is worth noting that the distinct profile of BM immune composition does not appear to impact infant growth or predisposition to infections. However, specific associations have been found with certain immune components. Therefore, further studies with a larger sample size and longer follow-up are needed to make more robust conclusions.

## 5 Conclusion

Two different BM immune profiles among Mediterranean mothers have been described. This study highlights the importance of the time of sampling of lactation when analyzing BM composition since the BM immunotypes can only be observed after the global analysis of the two sampling points. The results characterizing the BM immunotypes indicate that not all the mothers have the same evolution in terms of BM immune components from day 7 to day 15, except for IgA and IgM, which always had higher levels early in life (Figure 7).

The dynamically composed changes could be maternal-specific since we found differences in parity and exclusive breastfeeding and could induce different infant growth (Figure 7). It would be of great interest to add more sampling times to the study to follow-up on the BM evolution and to find out whether the different dynamic profiles are associated with infant characteristics because having only two sampling time points could be insufficient to draw strong conclusions about infant health later in life.

## Data availability statement

The raw data supporting the conclusion of this article will be made available by the authors, without undue reservation.

## Author contributions

MR-L, MCo, and FP-C: conceptualization. KR-A and AF-B: data curation, methodology, and formal analysis. KR-A: writing—original draft preparation. EV-L, AM-A, MCa, MS-R, CM-C, MR-L, MCo, and FP-C: writing—reviewing and editing. MCo and FP-C: funding acquisition. All authors contributed to the article and approved the submitted version.
