# Supplementary material for: Breast milk immune composition varies during the transition stage of lactation: characterization of immunotypes in the MAMI cohort
Source: Front Nutr. 2023 Nov 23;10:1252815. doi: 10.3389/fnut.2023.1252815 (PMC10702228; doi:10.3389/fnut.2023.1252815)
Supplement: Supplementary file 1 [file Table_1.docx]

Supplementary Material

Changes in breast milk immune components during the transition stage: characterization of immunotypes

Rio-Aige, K., Fernández-Bargalló, A., Vegas-Lozano, E., Miñarro-Alonso, A., Castell, M., Selma-Royo, M., Martínez-Costa, C., Rodríguez-Lagunas, M.J.*, Collado, M.C., Pérez-Cano, F.J.

*** Correspondence:** Corresponding Author: [mjrodriguez@ub.edu](mailto:mjrodriguez@ub.edu)

**Supplementary table 1.** Immune components in BM immunotypes at the two sampling time points.

|  | | BM-I, n = 39 | | BM-II, n = 36 | |
| --- | --- | --- | --- | --- | --- |
| **Immunoglobulins (mg/L)** | | **7d** | **15d** | **7d** | **15d** |
| **Total** |  | 1431.96 ± 92.20 | 905.72 ± 66.56 | 1264.19 ± 81.79 | 1032.80 ± 97.12 |
| **IgM** |  | 213.06 ± 58.89 | 111.84 ± 34.07 | 97.44 ± 26.07 | 185.97 ± 67.88 |
| **IgG** |  | 26.80 ± 2.50 | 5.61 ± 0.87 | 6.59 ± 1.37 * | 25.90 ± 4.10 * |
|  | **IgG1** | 14.67 ± 1.62 | 3.07 ± 0.55 | 2.99 ± 0.41 * | 17.20 ± 3.31 * |
|  | **IgG2** | 9.32 ± 0.87 | 2.04 ± 0.27 | 2.93 ± 0.81 * | 5.66 ± 0.49 * |
|  | **IgG3** | 1.42 ± 0.17 | 0.27 ± 0.04 | 0.47 ± 0.15 * | 1.87 ± 0.48 * |
|  | **IgG4** | 1.41 ± 0.19 | 0.23 ± 0.07 | 0.20 ± 0.03 * | 1.18 ± 0.16 * |
|  | **Th1** | 25.40 ± 2.37 | 5.38 ± 0.82 | 6.38 ± 1.35 * | 24.73 ± 4.06 * |
|  | **Th2** | 1.41 ± 0.19 | 0.23 ± 0.07 | 0.20 ± 0.03 * | 1.13 ± 0.16 * |
|  | **Th1/Th2** | 40.66 ± 6.85 | 38.91 ± 6.21 | 35.42 ± 3.98 * | 49.98 ± 9.62 * |
| **IgA** |  | 1192.09 ± 79.43 | 788.27 ± 55.14 | 1160.16 ± 77.71 | 820.92 ± 71.49 |
| **IgE** |  | 0.0063 ± 0.0005 | 0.0012 ± 0.0003 | 0.0012 ± 0.0001 * | 0.0038 ± 0.0003 * |
| **Cytokines (pg/mL)** | |  |  |  |  |
|  | **GM-CSF** | 0.20 ± 0.20 | 0.10 ± 0.10 | 1.94 ± 1.40 | 0.00 ± 0.00 |
|  | **IFN-γ** | 0.05 ± 0.03 | 1.39 ± 0.72 | 1.09 ± 0.45 | 0.71 ± 0.37 |
|  | **IL-1β** | 2.98 ± 1.13 | 0.56 ± 0.33 | 7.93 ± 4.81 | 16.55 ± 13.20 |
|  | **IL-2** | 0.90 ± 0.42 | 0.10 ± 0.10 | 2.19 ± 1.09 | 0.20 ± 0.15 |
|  | **IL-4** | 0.40 ± 0.27 | 0.00 ± 0.00 | 0.07 ± 0.06 | 0.02 ± 0.02 |
|  | **IL-5** | 0.24 ± 0.14 | 1.33 ± 0.86 | 1.11 ± 0.50 | 0.08 ± 0.06 |
|  | **IL-6** | 48.26 ± 28.16 | 8.41 ± 2.45 | 47.22 ± 10.06 | 53.73 ± 24.06 |
|  | **IL-9** | 0.41 ± 0.27 | 0.12 ± 0.06 | 0.89 ± 0.49 | 0.11 ± 0.11 |
|  | **IL-10** | 1.36 ± 1.10 | 0.42 ± 0.19 | 0.90 ± 0.40 | 0.84 ± 0.39 |
|  | **IL-12** | 0.65 ± 0.59 | 0.16 ± 0.03 | 0.21 ± 0.09 | 0.05 ± 0.02 |
|  | **IL-13** | 0.00 ± 0.00 | 0.03 ± 0.03 | 0.09 ± 0.09 | 0.03 ± 0.03 |
|  | **IL-17** | 0.14 ± 0.08 | 0.17 ± 0.11 | 1.08 ± 0.49 | 0.16 ± 0.11 |
|  | **IL-18** | 7.54 ± 1.11 | 32.76 ± 9.84 | 26.22 ± 5.89 * | 19.24 ± 5.94 |
|  | **IL-21** | 17.66 ± 6.67 | 6.39 ± 1.62 | 4.65 ± 0.76 | 5.03 ± 2.09 |
|  | **IL-22** | 5.54 ± 1.61 | 9.97 ± 2.00 | 10.63 ± 1.38 * | 4.24 ± 1.15 * |
|  | **IL-23** | 0.95 ± 0.41 | 0.00 ± 0.00 | 0.00 ± 0.00 | 0.29 ± 0.16 |
|  | **IL-27** | 0.16 ± 0.16 | 3.49 ± 3.49 | 0.41 ± 0.41 | 0.00 ± 0.00 |
|  | **TNF-α** | 2.80 ± 0.57 | 1.52 ± 0.34 | 2.35 ± 0.70 | 1.85 ± 0.22 |
| **Adipokines (pg/mL)** | |  |  |  |  |
|  | **Leptin** | 403.12 ± 71.05 | 827.21 ± 125.85 | 790.83 ± 110.16 * | 445.45 ± 77.45 |
|  | **Adiponectin** | 13468.39 ± 1291.05 | 9891.13 ± 1069.99 | 12432.21 ± 1699.93 | 18155.15 ± 3467.48 * |
|  | **L/A ratio** | 0.05 ± 0.01 | 0.14 ± 0.03 | 0.10 ± 0.02 * | 0.03 ± 0.00 * |

Data shown are expressed as mean ± SEM. Multivariate GLM was conducted to analyze the influence of sampling time (Time) and clustering group (Group) and the interaction (Int) between them. p values derived from the multivariate GLM (Group, Time and Int) are represented below. For those components in which the interaction between group and time was significant, indicating that the temporal evolution differs between groups, the Student's t-test was performed while keeping the time variable constant. The p-values were adjusted for multiple comparisons using the False Discovery Rate (FDR) correction. **p* < 0.05.

| p value | | | | |
| --- | --- | --- | --- | --- |
| **Immunoglobulins (mg/L)** | | **Group** | **Time** | **Int** |
| **Total** |  | 0.246 | 0.002 | <0.0001 |
| **IgM** |  | 0.186 | 0.045 | 0.951 |
| **IgG** |  | 0.591 | 0.115 | <0.0001 |
|  | **IgG1** | 0.688 | 0.824 | <0.0001 |
|  | **IgG2** | 0.046 | 0.001 | <0.0001 |
|  | **IgG3** | 0.479 | 0.619 | <0.0001 |
|  | **IgG4** | 0.449 | 0.473 | <0.0001 |
|  | **Th1** | 0.686 | 0.106 | <0.0001 |
|  | **Th2** | 0.449 | 0.473 | <0.0001 |
|  | **Th1/Th2** | 0.707 | 0.311 | <0.007 |
| **IgA** |  | 0.724 | <0.0001 | 0.945 |
| **IgE** |  | <0.0001 | <0.0001 | <0.0001 |
| **Cytokines (pg/mL)** | |  |  |  |
|  | **GM-CSF** | 0.254 | 0.062 | 0.101 |
|  | **IFN-γ** | 0.300 | 0.320 | 0.028 |
|  | **IL-1β** | 0.091 | 0.108 | 0.302 |
|  | **IL-2** | 0.458 | 0.014 | 0.732 |
|  | **IL-4** | 0.430 | 0.088 | 0.280 |
|  | **IL-5** | 0.699 | 0.320 | 0.040 |
|  | **IL-6** | 0.011 | 0.018 | 0.455 |
|  | **IL-9** | 0.538 | 0.084 | 0.279 |
|  | **IL-10** | 0.275 | 0.462 | 0.873 |
|  | **IL-12** | 0.469 | 0.456 | 0.291 |
|  | **IL-13** | 0.419 | 0.936 | 0.421 |
|  | **IL-17** | 0.097 | 0.072 | 0.873 |
|  | **IL-18** | 0.994 | 0.121 | 0.000 |
|  | **IL-21** | 0.170 | 0.342 | 0.780 |
|  | **IL-22** | 0.893 | 0.650 | 0.000 |
|  | **IL-23** | 0.224 | 0.224 | 0.003 |
|  | **IL-27** | 0.533 | 0.994 | 0.341 |
|  | **TNF-α** | 0.666 | 0.237 | 0.003 |
| **Adipokines (pg/mL)** | |  |  |  |
|  | **Leptin** | 0.450 | 0.857 | <0.0001 |
|  | **Adiponectin** | 0.154 | 0.686 | 0.003 |
|  | **L/A ratio** | 0.131 | 0.696 | <0.0001 |


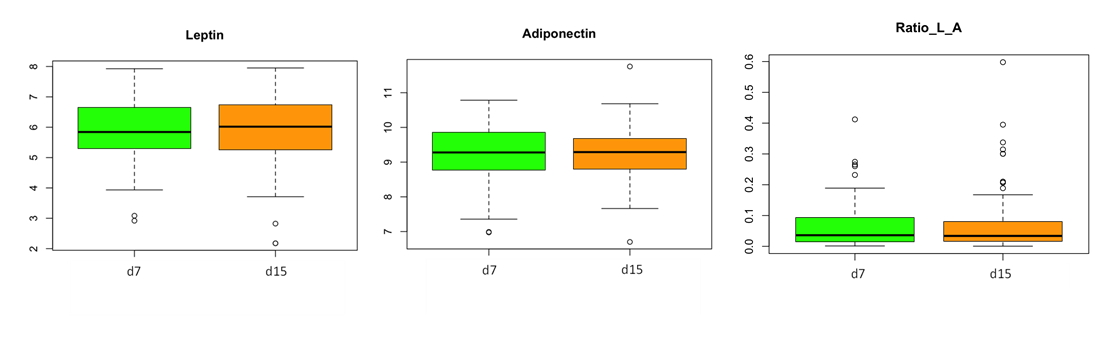


**Suplementary figure 1**. Concentration of leptin and adiponectin levels and the ratio of them in milk at day 7 and at day 15 from the same mothers (N=75). The concentration of each analysis (pg/mL) was normalized logarithmically and the interquartile range is expressed in box plots. Student’s t-test was used to determine significant differences between sampling days. The p-values were adjusted for multiple comparisons using the False Discovery Rate (FDR) correction. **p* < 0.05.

**Supplementary Figure 2.** Classification of the functions of cytokines. [1,2,11,3–10]

1. Espinosa-Martos, I.; Montilla, A.; Gómez De Segura, A.; Escuder, D.; Bustos, G.; Pallás, C.; Rodríguez, J.M.; Corzo, N.; Fernández, L. Bacteriological, biochemical, and immunological modifications in human colostrum after holder pasteurisation. *J. Pediatr. Gastroenterol. Nutr.* **2013**, *56*, 560–568, doi:10.1097/MPG.0b013e31828393ed.

2. Aparicio, M.; Browne, P.D.; Hechler, C.; Beijers, R.; Rodríguez, J.M.; de Weerth, C.; Fernández, L. Human milk cortisol and immune factors over the first three postnatal months: Relations to maternal psychosocial distress. *PLoS One* **2020**, *15*, e0233554, doi:10.1371/journal.pone.0233554.

3. Ballard, O.; Morrow, A.L. Human milk composition: nutrients and bioactive factors. *Pediatr. Clin. North Am.* **2013**, *60*, 49–74, doi:10.1016/j.pcl.2012.10.002.

4. Aparicio-Siegmund, S.; Garbers, C. The biology of interleukin-27 reveals unique pro- and anti-inflammatory functions in immunity. *Cytokine Growth Factor Rev.* **2015**, *26*, 579–586, doi:10.1016/j.cytogfr.2015.07.008.

5. Hawkes, J.S.; Bryan, D.L.; James, M.J.; Gibson, R.A. Cytokines (IL-1β, IL-6, TNF-α, TGF-β1, and TGF-β2) and prostaglandin E2 in human milk during the first three months postpartum. *Pediatr. Res.* **1999**, *46*, 194–199, doi:10.1203/00006450-199908000-00012.

6. Santarlasci, V.; Cosmi, L.; Maggi, L.; Liotta, F.; Annunziato, F. IL-1 and T helper immune responses. *Front. Immunol.* **2013**, *4*, 182, doi:10.3389/fimmu.2013.00182.

7. Ross, S.H.; Cantrell, D.A. Signaling and Function of Interleukin-2 in T Lymphocytes. *Annu. Rev. Immunol.* **2018**, *36*, 411–433, doi:10.1146/annurev-immunol-042617-053352.

8. Garofalo, R.; Chheda, S.; Mei, F.; Palkowetz, K.H.; Rudloff, E.; Schmalstieg, F.C.; Rassin, D.K.; Goldman, A.S.; Utziversity, T. Interleukin-10 in Human Milk. **1995**, *37*, 444–449.

9. Saso, A.; Blyuss, O.; Munblit, D.; Faal, A.; Moore, S.E.; Le Doare, K. Breast milk cytokines and early growth in Gambian infants. *Front. Pediatr.* **2019**, *6*, 414, doi:10.3389/fped.2018.00414.

10. Heinzerling, N.P.; Donohoe, D.; Fredrich, K.; Gourlay, D.M.; Liedel, J.L. Interleukin-23 Increases Intestinal Epithelial Cell Permeability in Vitro. *Eur. J. Pediatr. Surg.* **2016**, *26*, 260–266, doi:10.1055/s-0035-1551563.

11. Kubota, T.; Shimojo, N.; Nonaka, K.; Yamashita, M.; Ohara, O.; Igoshi, Y.; Ozawa, N.; Nakano, T.; Morita, Y.; Inoue, Y.; et al. Prebiotic consumption in pregnant and lactating women increases IL-27 expression in human milk. *Br. J. Nutr.* **2014**, *111*, 625–632, doi:10.1017/S0007114513003036.

**Supplementary Figure 3**. Concentration of cytokines associated responses in breast milk at day 7 and at day 15 from the same mothers (N=75). The concentration of each analysis (pg/mL) was normalized logarithmically, and the interquartile range is expressed in box plots. Student’s t-test was used to determine significant differences between sampling days. The p-values were adjusted for multiple comparisons using the False Discovery Rate (FDR) correction. **p* < 0.05.


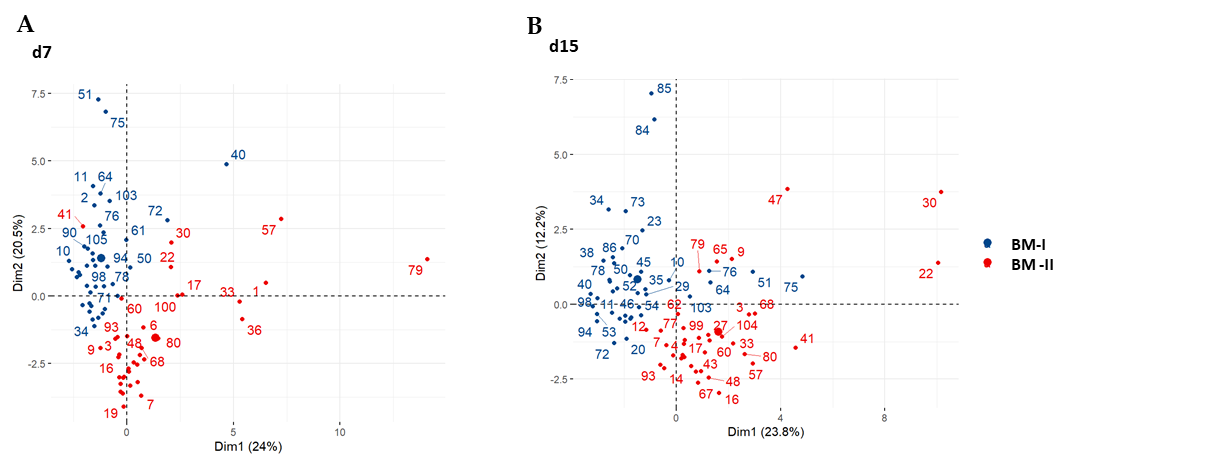


**Supplementary Figure 4.** Multiple Factor Analysis (MFA) for the concentration of immune factors in the breast milk of 75 mothers (MAMI cohort) at day 7 (A) and at day 15 (B). In blue the mothers who belong to breast milk immunotype I (BM-I), in red the mothers who belong to breast milk immunotype II (BM-II).

**Supplementary Figure 5.** Mixed Graphical Model (MGM) representing conditional dependencies directly related with the breast milk (BM) immunotype group. Nodes (vertices) represent variables and edges represent conditional dependencies. Red edges represent a negative correlation between multinomial variables; green edges represent a positive correlation between multinomial variables; grey edges represent a relation with a categorical variable (A). Factor Map indicating the main variables in each dimension and their correlations. The longer the vector, the more force it has in the dimension. The closer two vectors are, the more correlated they are (B).

**Supplementary Figure 6.** Multiple Factor Analysis (MFA) for the concentration of immune factors in the human milk of 75 mothers (MAMI cohort) taking into account the two sampling days regarding the secretor gene (A) or diet (B). In panel A, mothers with the secretor gene are depicted in blue, in red those without the secretor gene, and in grey without classification. In panel B, mothers depicted in blue belong to Diet II group and in red to Diet I groups. The intake of total of polyphenols (C) and Vitamin D (D) by the mothers belonging to the two clusters, breast milk immunotype I (BM-I) and breast milk immunotype II (BM-II) are shown. Student’s t-test was used to determine significant differences between groups. The p-values were adjusted for multiple comparisons using the False Discovery Rate (FDR) correction. **p* < 0.05.

**
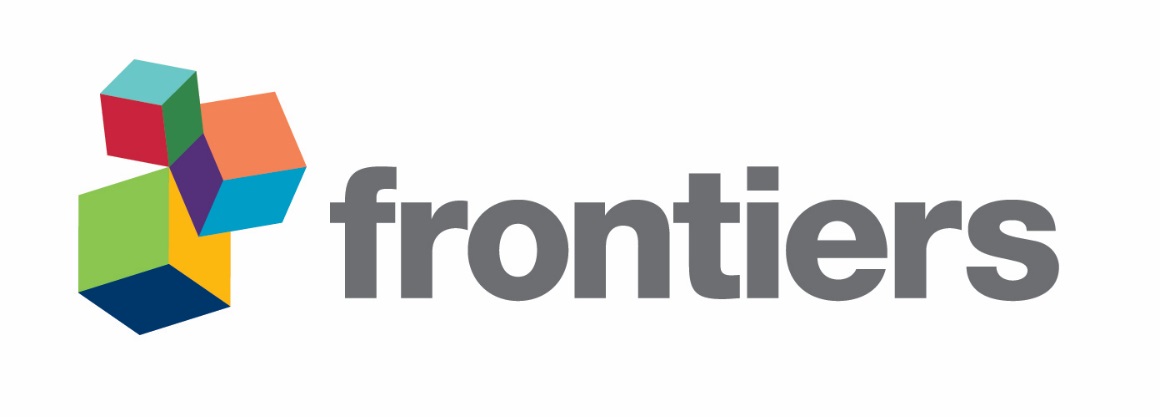
**
